# Knowledge About HIV/AIDS Among High School Students in Erbil City/Iraq

**DOI:** 10.5539/gjhs.v7n1p16

**Published:** 2014-07-29

**Authors:** Samir M. Othman

**Affiliations:** 1 College of Medicine, Hawler Medical University, Erbil, Iraq

**Keywords:** knowledge, HIV/AIDS, high school students, socio-demographic, Erbil, Iraq

## Abstract

**Background and Objectives:**

HIV/AIDS is as a major public health problem which leads to serious challenges to humankind globally. The aim of this study was to assess the knowledge about HIV/AIDS among high school students in Erbil city and to investigate the association between high school students’ socio-demographic characteristics and their level of knowledge about HIV/AIDS.

**Methods:**

This descriptive cross-sectional study was carried out in three high schools in Erbil city from February to April 2014. A sample of 437 students was included in the study from fourth, fifth and sixth stages. A multistage cluster sampling method was used to select the students. Data analysis included descriptive statistics and chi-square association test for categorical variables.

**Results:**

The age range of the students was between 14 and 21 years with mean ± standard deviation of 16.0 ± 0. 927 years. All the students had heard about AIDS where around two thirds of students had heard from mass media like TV/Radio. Around 45% of students had good knowledge scores about HIV/AIDS, and 43.7% had acceptable knowledge scores, while only 11.2% had poor knowledge scores. There was a statistically significant association between high knowledge score about HIV/AIDS with older age, male gender, and typical school type (P < 0.001). High socio-economic status of students was significantly associated with high score of knowledge about HIV/AIDS (P = 0.005).

**Conclusion:**

The overall rate of knowledge (acceptable and good) about HIV/AIDS among high school students was high. Socio-demographic characteristics of students have an effect on their knowledge about HIV/AIDS.

## 1. Introduction

The Acquired Immunodeficiency Syndrome (AIDS) is one of the most complex health problems of the 21^st^ century. Since its appearance in 1981, the HIV/AIDS pandemic, which leads to a serious challenge to humankind, without doubt, has become one of the most serious infectious diseases (Taher& Abdelhai, 2011). HIV infection, which has increased among young people who aged 15–24 years, is a development problem. Recent UNAIDS report on the global AIDS epidemic estimates that there were 35.3 million [32.2 million–38.8 million] people living with HIV. In 2012 worldwide, 2.3 million [1.9 million–2.7 million] people became newly infected with HIV, and 1.6 million [1.4 million–1.9 million] people died from AIDS-related causes (UNAIDS, 2013). It also declares that approximately 0.8% of adults aged 15–49 years worldwide are living with HIV (UNAIDS, 2012a). Over the past decades, adolescent productive and sexual health concerns have increasingly been on national agendas. For many countries, this concern has been driven by the high prevalence of HIV/AIDS among young people (CDC, 2007).

Globally, it is known that there is a lack of HIV knowledge among youth between the ages of 15–24. The WHO stated that youth are at the core of preventing the progression of the HIV/AIDS pandemic. The WHO estimates that youth aged 15–24 comprise 50% of all new HIV infections and consequently must be targeted for education in decreasing transmission and reducing the stigmatization of an HIV diagnosis (WHO, 2004).

Iraq is still among the low prevalence countries for HIV/AIDS. In the period from 1986 to December 2011, 615 HIV cases were officially reported: 309 of which (50%) were Iraqi citizens, and 59 of which are still alive. The distribution of the cases is as follows; 85% males and 15% females; 66% were hemophilic by parental route; 17% by heterosexual route; 5% vertical transmission from infected mothers (Alwan, 2004; UNAIDS, 2012b).

During the period of March 2003 to December 2011, after changing the political regimen in Iraq, 131 new HIV cases were reported, 51 (39%) of them were Iraqi citizens. Within this period, the transmission route shifted towards the heterosexual route, as the government has put in place strict blood safety measures (UNAIDS, 2012b).

Many factors put developing countries (like Iraq) at greater risk for developing HIV. Examples for these factors illiteracy, low per capita income, gender discrimination, poor knowledge about routes of transmission. Social stigma might disallow people with risky behaviors from seeking HIV testing or disclosing a positive status. Population growth, migration to urban areas, sociocultural barriers and poor prevention efforts might also contributing to the spread of HIV/AIDS (UNAIDS, 2012a). Immigration of different kinds of people, especially from low income countries, who come to the region to work in infrastructure development, now is very common. This might increases the possibility of spreading HIV between them, and thereby to the inhabitants.

Insufficiency of basic knowledge regarding modes of transmission of HIV, and fear of knowing one’s HIV status can lead to denial one’s risk of contracting HIV and failure to get tested. Many HIV positive youth in the USA do not know that they are infected (WHO, 1988).

High school students’ knowledge about HIV/ AIDS might be varies by their socio-demographic characteristics such as age, gender, school type, family head income, fathers’ and mothers’ educational levels, and source of information. The study of knowledge of HIV/AIDS is considered a basic first step in the education process and prevention of the disease (Huda & Amanullah, 2013).

There is no study in the region that highlights this situation. Therefore, this study aimed to assess the knowledge of high school students about HIV/AIDS in Erbil city and to find out associations between knowledge of students with certain socio-demographic characteristics.

## 2. Subjects and Methods

This descriptive cross-sectional study was carried out from February to April 2014 among students from three high schools (two public and one typical school) in Erbil city. Typical schools are also public schools that admit talented students and have special focused study programs that are entirely in English language. A sample of 437 students was included in the study from fourth, fifth and sixth stages. A multistage cluster sampling method was used to selecting the students. From the lists of all high schools of Erbil city that was obtained from the Directorate of Education, we selected randomly two schools from public and one from the typical schools. The classes were identified by the administrative department of each school. In each school one to two classes from each of the three stages were randomly selected and all the students in the selected classes were invited to participate in the study.

Data were collected by the researcher using a simplified, structured, self-administered questionnaire filled in by students at their class. At each class the students were invited to participate in the study at the beginning of each session after explaining the purpose of the study with the assistance of the teacher. Permission was obtained for students to take 10–15 minutes at the beginning of the session to complete the questionnaire. Students were informed not to discuss the questions with each other. If they had any queries, they were encouraged to ask a researcher.

At the end of the session, the researcher checked all questionnaires that had been answered and returned any questionnaires with missing information for the students to complete. The modified questionnaire used for this purpose was based on the World Health Organization AIDS programme knowledge, attitudes, beliefs and practices survey in 1988, and on other literatures (WHO, 2000; Xinming, 2003). Two PhD holders whose mother tongue is Kurdish (the local language) and their language of study during under and post graduate studies was English. The first one translated the questionnaire from English to Kurdish and the second one made reverse translation from Kurdish to English and then the two English versions were compared and found comparable.

The questionnaire started by outlining the benefits and purposes of the study and composing of three parts; first part including the socio-demographic characteristics of the sample such as (age, sex, marital status, name of school, stage, educational level of father and mother, occupation of father and mother, family head income and home ownership). The second part of questionnaire included the sources of information about HIV/AIDS, and third part included question related to knowledge about HIV/AIDS, e.g. causative agent, modes of transmission and knowledge about sequence of HIV infection and prevention.

Students were asked to mark the correct answer for each question (yes, no or don’t know). Each correct answer was given a score of ‘one’ and each wrong answer was given a score of ‘zero’. The knowledge was categorized into; poor knowledge (0-6score), acceptable knowledge (7-12 score) and good knowledge (13-18 score). The total scores for each student therefore ranged from 0–18.

Regarding socio-economic status of participants; six parameters were used to score. Each parameter was scored as follows; educational level of mother and father; illiterate = 0, primary school = 1, intermediate school = 3, secondary school = 4 and institute/college = 5, occupation of mother and father; employed = 2, un employed=0, family head income; < 500,000ID = 0, 500,000-1,000,000ID = 1, > 1,000,000ID = 2, home ownership; rented = 0, owned = 1. By summarizing scores of the six parameters, the socio-economic level was stratified into: Low (0–5), Medium (6–10), High (11–15).

The study protocol was approved by the Scientific and Ethical Committee of College of Medicine of Hawler Medical University and an informed consent was obtained from all students prior to participation in the study. All selected students were cooperative and agreed to participate in the study.

### 2.1 Data Analysis

The Statistical Package for Social Sciences (SPSS, Chicago, IL, USA, version 18) was used for data entry and analysis. Two approaches were used; descriptive and analytic. The descriptive approach included calculation of frequencies, percentages, means, standard deviations, while in the second approach; Chi-square test of association was used to test the significant association between categorical variables. P value of ≤ 0.05 was considered as statistically significant.

## 3. Results

### 3.1 Socio-Demographic Characteristics of Study Sample

This study involved 437 high school students (213 from typical and 224 from public schools); 221 (50.6%) males and 216 (49.4%) females, with male: female ratio of 1.02:1.0. The students’ age ranged between 14 and 21 years with mean ± standard deviation of 16.0 ± 0.927, and only 17 (3.9%) were married. Regarding parents’ education; more than two thirds of fathers and half of mothers were graduate secondary school and higher level, while illiteracy rate was only 4.5% among fathers in comparison to 16.5% among mothers. Details of socio-economic status illustrated in (Table 1).

**Table 1:** Distribution of sample by parents’ socio-economic status

Variables	No.	% (N=437)
**Fathers’ education**		
**Illiterate**	20	4.57
**Primary**	46	10.52
**Intermediate**	69	15.80
**Secondary**	120	27.45
**Institutes/College**	182	41.64
**Mothers’ education**		
**Illiterate**	72	16.50
**Primary**	87	19.90
**Intermediate**	61	13.95
**Secondary**	88	20.13
**Institutes/College**	129	29.50
**Fathers’ occupation**		
**Employed**	399	91.3
**Un employed**	38	8.70
**Mothers’ occupation**		
**Employed**	194	44.4
**Un employed (housewife)**	243	55.6
**Family head income ID/month**		
**<500,000**	38	8.7
**500,000-1,000,000**	161	36.8
**>1,000,000**	238	54.5
**Home ownership**		
**Owned**	404	92.4
**Rented**	33	7.6

### 3.2 Source of Information About HIV/AIDS

All students had heard about AIDS. As illustrated in Table 2, around two thirds of students had heard from mass media like TV/Radio (63%) and newspaper (49%).

**Table 2:** Source of information about HIV/AIDS

Heard about HIV/AIDS from	No.	%
TV/Radio	275	62.9
Newspapers	210	49.0
Teachers from school	214	48.1
Friends/Relatives	191	43.7
Family (parents, sibling’s)	133	30.4

### 3.3 Knowledge of Students About Modes of Transmission and Consequence of HIV/DIDS

The vast majority (92.2%) of students knew that HIV/AIDS is a viral disease. Accordingly, 94.3% of students knew that HIV can be transmitted through sexual intercourse. The majority of students were also aware that HIV can be transmitted through blood transfusions (83.5%), from mother to child (75.3%), and through sharing needles or syringes (73.7%). However, there was confusion about some routes of transmission. For example, only 56.8% and 54.2% of the students correctly answered that social kissing and shaking hands with HIV patients don’t spread HIV, respectively. Nearly half of students knew that HIV could not be transmitted by wearing the same clothes of infected patients and through swimming pools. Regarding knowledge of students about certain consequences of HIV/AIDS, only 12.8% and 21.7% of students correctly answered that all HIV/AIDS patients will eventually die and there is no specific therapy for HIV/AIDS treatment, respectively (Table 3).

**Table 3:** Knowledge of students regarding routes of transmission and consequences of HIV/AIDS

Questions with correct answer	The correct answer	

	No.	%
HIV/AIDS can be transmitted by sexual intercourse (yes)	412	94.3
HIV/AIDS can be transmitted by blood transfusion (yes)	365	83.5
HIV/AIDS can be transmitted from mother to child (yes)	329	75.3
HIV/AIDS can be transmitted by breast feeding (yes)	210	48.1
HIV/AIDS can be transmitted by sharing needles (yes)	322	73.7
HIV/AIDS can be transmitted by sharing razors (yes)	218	49.9
HIV/AIDS can be transmitted by sharing toothbrush (yes)	193	44.2
HIV/AIDS can be transmitted by cough and sneezing (no)	116	26.5
HIV/AIDS can be transmitted in swimming pools (no)	123	28.1
HIV/AIDS can be transmitted by social kissing (no)	124	28.4
HIV/AIDS can be transmitted by shaking hand (no)	131	30.0
HIV/AIDS can be transmitted by wearing clothes of infected person with HIV/AIDS (no)	322	73.7
**Knowledge of students about consequences of HIV/AIDS**	
HIV/AIDS, infection remains for long life (yes)	242	55.4
Do all HIV/AIDS patients will eventually die? (yes)	56	12.8
Is there a specific therapy for AIDS treatment? (no)	95	21.7
Is there vaccine for protection? (no)	295	67.5

### 3.4 Knowledge Scores of Students

**Figure 1:**
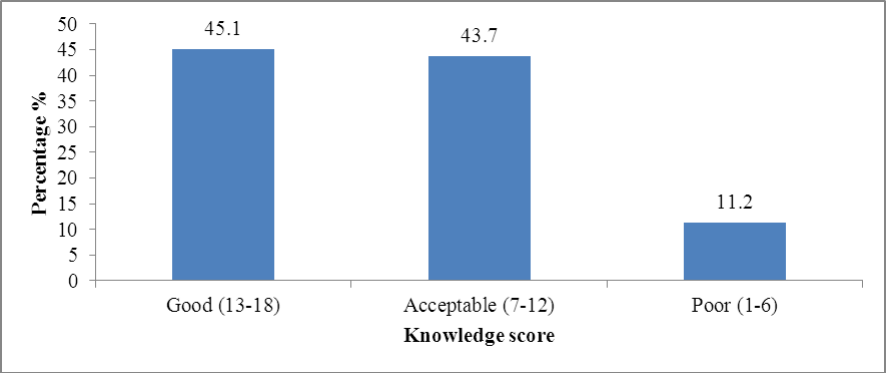
Overall Knowledge score among studied sample

Of 437 students, 197 (45.1%) had good knowledge scores, while 191 (43.7%) had acceptable knowledge scores, and 49 (11.2%) had poor knowledge scores about the HIV/AIDS (Figure 1).

### 3.5 Association Between Knowledge and Certain Demographic Characteristics of Students

A statistically significant association was found between knowledge levels and type of school, in which good level of knowledge was high among students from typical school, (P < 0.001). A statistically significant association between gender, age and knowledge was found in studied sample, in which good knowledge was higher among males and among age group more than 19 years (P < 0.001). On the other hand, there was also a statistically significant association between knowledge and socio-economic status of students, in which good knowledge was highest among students with high socio-economic status (P value < 0.001), (Table 4).

**Table 4:** Relationship between knowledge and socio-demographic characteristics of students

Variables	Knowledge						N=437	P

	Poor (1-6)		Acceptable (7-12)		Good (13-18)			

	No.	%	No.	(%)	No.	(%)		
**Type of School**							
Typical	0	0.00	76	35.7	137	64.3	213	<0.001
Public	49	21.9	115	51.3	60	26.8	224	
**Age in years**							
<15	15	37.5	17	42.5	8	20.0	40	<0.001
15-18	32	10.0	138	43.0	151	47.0	321	
≥19	2	2.60	36	47.4	38	50.0	76	
**Gender**							
Male	11	5.0	97	43.9	113	51.1	221	<0.001
Female	38	17.6	94	43.5	84	38.9	216	
**Marital status**							
Single	47	11.2	186	44.3	187	44.5	420	0.455
Married	2	11.8	5	29.4	10	58.8	17	
**Socio-economic status**							
Low	11	20.8	26	49.1	16	30.2	53	0.005
Medium	24	14.2	74	43.8	71	42.0	149	
High	14	6.50	91	42.3	110	51.2	215	

### 3.6 Association Between Socio-Economic Status of Students and Type of School

There was a statistically significant association between socio-economic status of students and type of school, p value < 0.001, (Table 5).

**Table 5:** Association between socio-economic status of students and type of school

Type of School	Socio-economic status scores						N=437	P

	High		Medium		Poor			

	No.	%	No.	%	No.	%		
Typical	140	65.7	59	27.7	14	6.60	213	<0.001
Public	75	33.5	110	49.1	39	17.4	224	

## 4. Discussion

Adolescents regarded as high risk group for HIV/AIDS infection, some of them may have insufficient knowledge about the disease. The large populations of young adults in the communities with high levels of close social contact might have the potential to become the focus of epidemic. Knowledge is very useful tools prior to any intervention to assess the extent to which individuals or communities are in a position to adopt risk-free behaviors (Al-Qaseer & Al-Jawhari, 2004).

In this study all students (100%) had heard about HIV/AIDS, which is higher than that reported among students in Jordanian 97.1%, (Al-Qaseer & Al-Jawhari, 2004), Malaysian young adults, 95.7% (Wong et al., 2008) and Nigerian adolescents, 93% (Oyo-Ita et al., 2005). This might be attributed to variance in the time, that people now a day knows more about various diseases including AIDS.

The current study revealed that the main sources of information about HIV/AIDS was the mass media, with television/radio 62.9% ranking the first, followed by newspaper 49%. These findings are in agreement with studies done in Bangladesh (Huda & Amanullah, 2013), Nigeria (Asekun-Olarinmoye et al., 2011) and in India (Lal et al., 2000). The second important source of HIV/AIDS information was teachers from school 49%, friends 43.7% and family (parents/siblings) only 30.4%.

The result of this study recommended that a great emphasis should be paid to AIDS related information in mass media and involving all parents, teachers, relatives and students in AIDS education program.

This study showed that about 45% had a good knowledge about HIV/AIDS. This finding is somewhat consistent with the findings reported by Uddin et al. (Uddin et al., 2010), Huda, and Amanullah, on Bangladeshi adolescents (Nazmul & Amanullah, 2013) and UNESCO on the students in Cameroon (UNESCO, 2007).

By contrast, this finding is incompatible with the findings reported by Shirin and Ahmed in Dhaka City (Shirin & Ahmed, 2007) and Amanullah and Huda in Bangladesh (Amanullah & Huda, 2012). According to their findings, very few students were found to have a good knowledge about HIV/AIDS.

The current study showed that knowledge about HIV/AIDS was significantly associated with certain socio-demographic characteristics of students, and the results also showed that older and male students had higher level of knowledge about HIV/AIDS. This finding was compatible with the results of the report of Ministry of Health and Family Welfare, Bangladesh (MOH & FW, 2007) and results of the study conducted in Bangladesh (Uddin et al., 2010). The significant association between age and male students and high knowledge about HIV/AIDS in this study might be due to that older and male students desire to learn about the taboo subject and this might encouraged them to discuss HIV/AIDS with each other.

The results of this study revealed that students of typical school and those having high socio-economic status had high knowledge about HIV/AIDS and these associations were somewhat in agreement with the findings of a study done in Kerala in India (Lal et al., 2000) and in Malaysia (Tee & Huang, 2009). This may be due to that student of typical school and those having higher socio-economic status have higher degree of exposure to mass media including television, newspapers and internet. Furthermore, the quality of typical school, talented students in typical school and the higher educational level and economic status of their parents as revealed by this study might have positive relationship with having knowledge about HIV/AIDS. This finding is consistent with a study done in Sub-Saharan Africa (Jung et al., 2013).

### 4.1 Limitation of the Study

The researcher was restricted in asking questions concerning students’ sexual beliefs and behaviors in the questionnaire, because of conservative and religious society of the region. Furthermore, the results of this study can be serving as base information for future studies among youths. And the author suggested further study in future regarding health education intervention of HIV/AIDS among school children for longitudinal study.

## 5. Conclusions

The overall rate of knowledge (acceptable and good) about HIV/AIDS among high school students was high. The main source of information about HIV/AIDS was mass media. Typical school, male students, older students, students of high socio-economic status, have significantly highest degree of knowledge about HIV/AIDS.
